# Supplementary material for: Development of chemokine network inhibitors using combinatorial saturation mutagenesis
Source: Commun Biol. 2025 Apr 3;8:549. doi: 10.1038/s42003-025-07778-6 (PMC11969024; doi:10.1038/s42003-025-07778-6)
Supplement: Supplementary file 4 — Supplementary Data 2 [file 42003_2025_7778_MOESM4_ESM.zip › SupplementaryData3/scripts/xattune_dr.tar!/./script/analysis/JK/JK_E5_CCL5_THP1_plot.pdf]

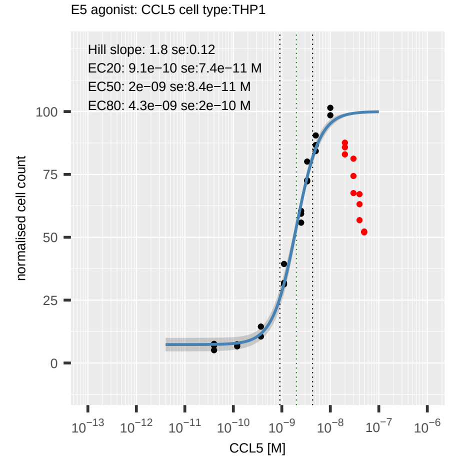

E5 agonist: CCL5 cell type:THP1
Hill slope: 1.8 se:0.12
EC20: 9.1e−10 se:7.4e−11 M
EC50: 2e−09 se:8.4e−11 M
EC80: 4.3e−09 se:2e−10 M
normalised cell count
100
75
50
25
0
10^−13
10^−12
10^−11
10^−10
10^−9
10^−8
10^−7
10^−6
CCL5 [M]
